# Complete chloroplast genome sequence of *Pinus tabuliformis* var. *henryi* (Mast.) C.T.Kuan 1983 (Pinaceae)

**DOI:** 10.1080/23802359.2023.2301013

**Published:** 2024-01-11

**Authors:** Xi Wang, Lin Zhao, Xing-Xue Yang, Zhan-Lin Liu

**Affiliations:** College of Life Sciences, Northwest University, Xi’an, China

**Keywords:** Complete chloroplast genome, phylogeny, Pinaceae, *Pinus tabuliformis* var. *henryi* (Mast.) C.T.Kuan 1983

## Abstract

*Pinus tabuliformis* var. *henryi* (Mast.) C.T.Kuan 1983 is an endemic and rare subtropical pine, mainly distributed in central China. In this study, we sequenced the complete chloroplast (cp) genome of *P. tabuliformis* var. *henryi* and reported it for the first time. The cp genome was 119,634 base pairs (bp) in total length, including two inverted repeats (IRs, 495 bp), separated by a large single-copy region (LSC, 65,600 bp) and a small single-copy region (SSC, 53,044 bp). There are 114 different genes in the cp genome of *P. tabuliformis* var. *henryi*, including 74 protein-coding genes, 36 transfer RNA genes, and four ribosomal RNA genes. The overall GC content of the cp genome was 38.5%. Our phylogenetic analysis of *P. tabuliformis* var. *henryi* demonstrated that it was closely related to *P. tabuliformis* and could be used to identify and analyze its genetic diversity, which was expected to provide new data for taxonomic and phylogenetic studies of Pinus.

## Introduction

*Pinus tabuliformis* var. *henryi* (Mast.) C.T.Kuan 1983 is a rare subtropical conifer species endemic to China that is distributed on the southern slope of the Daba Mountains and the branches of the Wuling Mountains in Central China’s provinces like Shaanxi, Sichuan, Chongqing, Hubei, and Hunan (Zhang 1990). It is an important and excellent species in oil, wood, afforestation, soil, and water conservation, as well as needle utilization, which has great economic value (Gernandt et al. 2005). The similarity of morphological features and the overlap of distributions with *Pinus tabuliformis* Carr. and *Pinus massoniana* Lamb. make it often regarded as a variant of other species (Fu et al. 1999). The long-standing taxonomic confusion made it deforested as other species with wide ranges and it has been listed on the ‘IUCN Red List of Threatened Species’ (Yang 2021). Some studies have found that compared with *P. tabuliformis*, *P. tabuliformis* var. *henryi* has a wider stem taper, smaller seeds (6.37 vs. 7.02 mm) and cones (4.44 vs. 5.58 cm), shorter (10.31 vs. 11.81 cm), narrower (1.21 vs. 1.24 cm), and thinner (0.70 vs. 0.79 mm) needles (Li and Xu 1989; Mao and Liu 1989). While the chloroplast (cp) genome is uniparentally inherited and conserved in structure, it has been widely used in molecular markers (Lee and Park 2021), barcode identification (Tang et al. 2022), phylogenetic analysis, and other fields (Wang et al. 2018). We determined the cp genome of *P. tabuliformis* var. *henryi* in this study, which is expected to contribute new data for the classification and phylogenetic study of *Pinus*.

## Materials and methods

**Figure 1.**
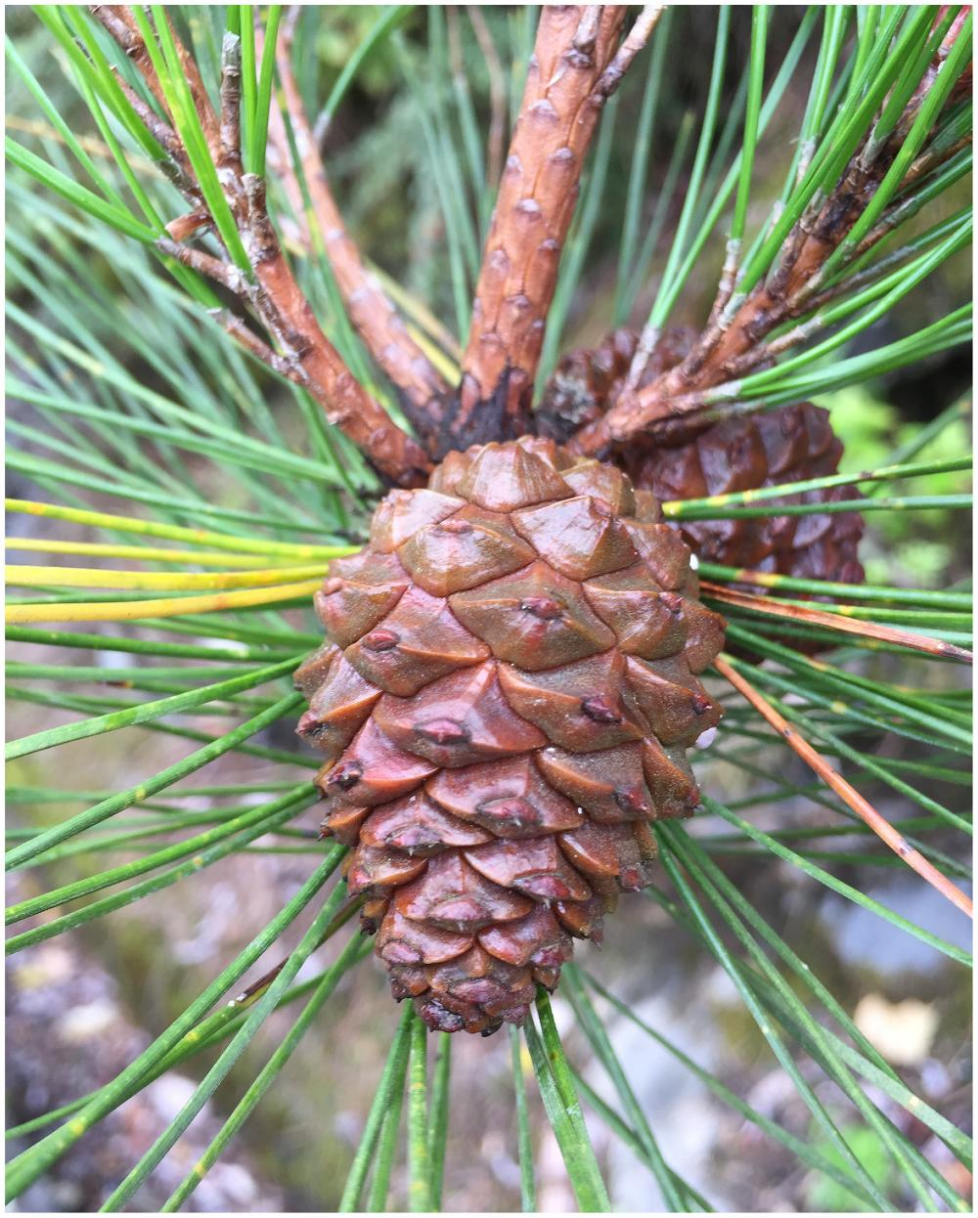
Plant image of *Pinus tabuliformis* var. *henryi*. This photo was taken by Zhan-Lin Liu on a hilltop in Wufeng County, Yichang City, Hubei Province, China. *P. tabuliformis* var. *henryi* is typically characterized by smaller fruits with no visible spines on the navel.

The leaves of *P. tabuliformis* var. *henryi* were collected from Hubei, China (31°71′97″ N, 109°58′53″ E), where they were also preserved in adherence to local regulations and were not subject to any particular limitations (Figure 1). In addition, the sampling site is open, and no special permission is required. The voucher (2018LIU151) was deposited at the Herbarium of Northwest University (Liuzl, liuzl@nwu.edu.cn). The improved CTAB method (Doyle 1987) was used for DNA extraction, and the Illumina HiSeq 2500 high-throughput sequencing platform was used to complete cp genome DNA sequencing, which was completed by Biomarker Technologies CO (Beijing, China). Initially, the NGS QC Toolkit V2.3.3 (Patel and Jain 2012) was utilized to filter all the raw reads. Subsequently, the processed double-ended clean reads were merged. The clean paired-end reads were then assembled using MIRA 4.0.2 (Chevreux et al. 2004), with the *P. tabuliformis* (KT740995) cp genome serving as a reference. We compared the assembly result with the reference sequence using MAFFT (Standley 2013), determined gene annotation using Geneious R9.0.2 (Masters et al. 2015), and performed further cp genome mapping using CPGView (Liu et al. 2022). Finally, the genome sequence has been submitted to NCBI (https://www.ncbi.nlm.nih.gov/) under accession number MW537659.

Moreover, the construction of the phylogenetic tree involved the utilization of 31 cp genomes from *Pinus*, with two taxa (*Larix sibirica* and *Larix kaempferi*) serving as outgroups. The complete dataset of *Pinus* cp genomes was aligned in MAFFT (Katoh and Standley 2013) and then compared using the Clustal W technique of MEGA version 11.0.13 and manually checked. Maximum-likelihood (ML) was then evaluated on the inferred evolutionary trees to achieve the best-fit model of cp genome sequence evolution for the model test version. The evolutionary tree was evaluated using 1000 bootstrap replicates, which were then used to approximate the ML tree branch support values. A ML for phylogenetic analysis was performed via IQ-Tree v1.6.10 (Nguyen et al. 2015) and visualized in FigTree v1.4.4 (http://tree.bio.ed.ac.uk/software/figtree).

## Results

**Figure 2.**
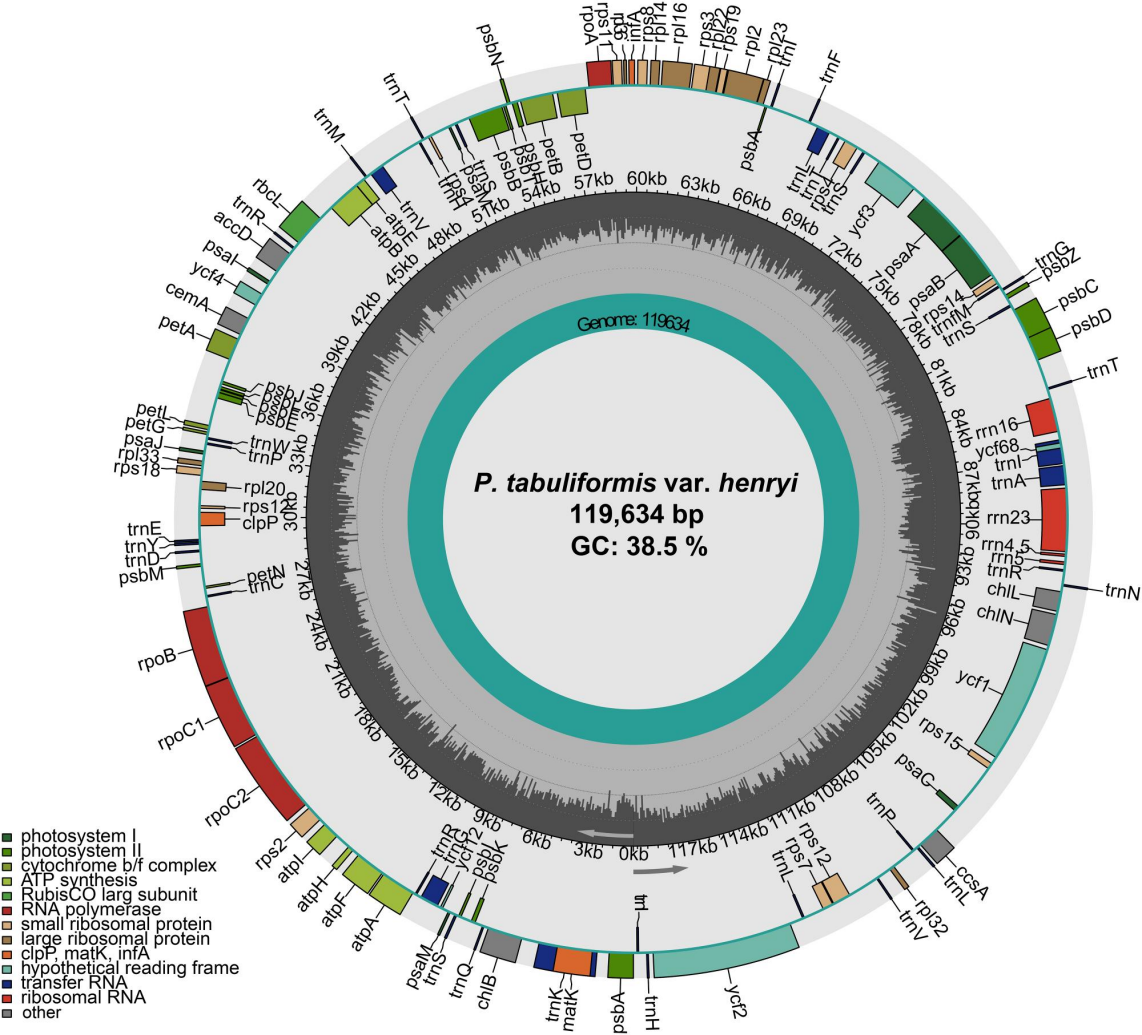
Chloroplast genome schematic map of *P. tabuliformis* var. *henryi*. The circle map of the complete chloroplast genome of *P. tabuliformis* var. *henryi*. Different colored boxes on the outer circle represent genes. The clockwise and counter-clockwise genes transcribed are drawn inside and outside of the circle, respectively. The black color region inside the inner circle indicates the GC content along the genome. In the bottom left corner, the map shows the key for the functional classification of the genes.

The results showed that the total length of the *P. tabuliformis* var. *henryi* cp genome was 119,634 bp with an average depth of 204× (Supplement Figure 1). The cp genome of *P. tabuliformis* var. *henryi* was 119,634 bp in length, with a large single-copy (LSC) region of 65,600 bp, a small single-copy (SSC) region of 53,044 bp, and a pair of inverted repeat (IR) regions of 495 bp (Figure 2). Moreover, we annotated the cp genome with a total of 114 genes, of which 108 were unique. These genes comprised 74 protein-coding genes (73 unique genes), 36 tRNA genes (31 unique genes), and four rRNA genes (four unique genes). The cp genome GC content was counted as 38.5%. While the introns were detected in 14 genes, of which 12 (*atpF*, *rpoC1*, *petB*, *petD*, *rp116*, *rp12*, *trnK-UUU*, *trnG-UCC*, *trnV-UAC*, *trnL-UAA*, *trnI-GAU*, and *trnA-UGC*) contained one intron, and two genes (*rps12*, *ycf3*) contained two introns. One of the trans-spliced genes, *rps12*, was detected by CPGView, which has three unique exons, two of which were duplicated (Supplement Figure 2).

**Figure 3.**
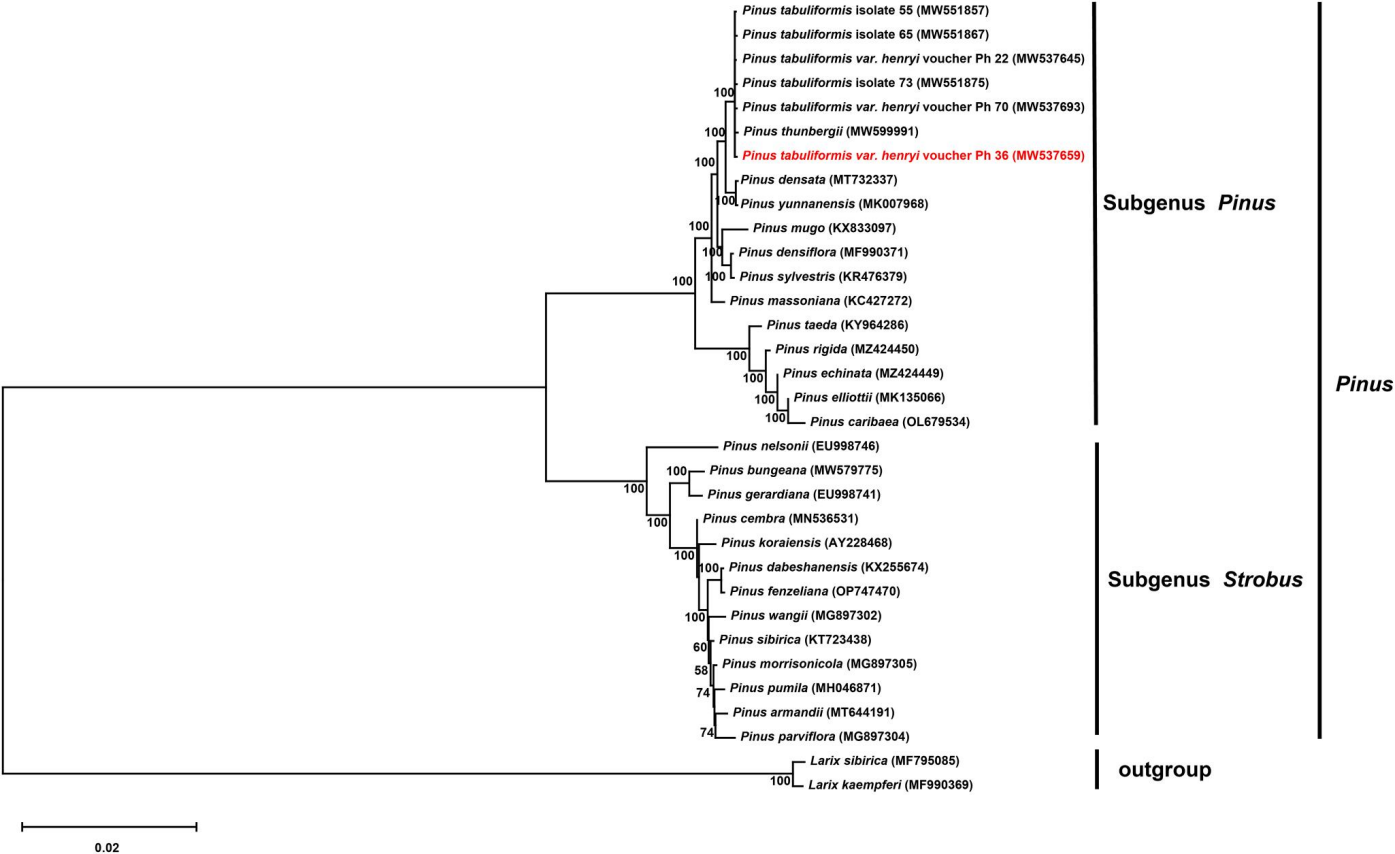
The phylogenetic position for *P. tabuliformis* var. *henryi* according to the ML (maximum-likelihood) phylogenetic tree was constructed based on 33 chloroplast genomes. Ultrafast bootstrap values are shown below the nodes, with 1000 bootstrap replicates. All the complete chloroplast genome sequences are available in GenBank. The following sequences were used: *Pinus tabuliformis* isolate 55 MW551857, *Pinus tabuliformis* isolate 65 MW551867, *Pinus tabuliformis* var. *henryi* voucher Ph 22 MW537645, *Pinus tabuliformis* isolate 73 MW551875, *Pinus tabuliformis* var. *henryi voucher Ph 70* MW537693, *Pinus thunbergii* MW599991 (Wakasugi et al. 1994), *Pinus densata* MT732337 (Li et al. 2021), *Pinus yunnanensis* MK007968, *Pinus mugo* KX833097 (Celiński et al. 2017), *Pinus densiflora* MF990371 (Xia et al. 2021), *Pinus sylvestris* KR476379, *Pinus massoniana* KC427272, *Pinus taeda* KY964286 (Asaf et al. 2018), *Pinus rigida* MZ424450 (Gernandt et al. 2018), *Pinus echinata* MZ424449, *Pinus elliottii* MK135066, *Pinus caribaea* OL679534, *Pinus nelsonii* EU998746 (Cronn et al. 2008), *Pinus bungeana* MW579775, *Pinus gerardiana* EU998741 (Cronn et al. 2008), *Pinus cembra* MN536531 (Schott et al. 2019), *Pinus koraiensis* AY228468, *Pinus dabeshanensis* KX255674 (Duan et al. 2016), *Pinus fenzeliana* OP747470, *Pinus wangii* MG897302, *Pinus sibirica* KT723438 (Baturina et al. 2019), *Pinus morrisonicola* MG897305, *Pinus pumila* MH046871 (Zeb et al. 2019), *Pinus armandii* MT644191 (Li et al. 2016), *Pinus parviflora* MG897304, *Larix sibirica* MF795085 (Bondar et al. 2019), and *Larix kaempferi* MF990369 (Kim et al. 2017).

To reveal the phylogenetic position of *P. tabuliformis* var. *henryi* with other members of *Pinus*, we performed a phylogenetic analysis based on 31 cp genomes of *Pinus* and two taxa (*Larix sibirica* and *Larix kaempferi*) as outgroups (Figure 3). Since the affinities of some species could not be clearly shown, we supplemented this by showing the affinities with the same length of branches (Supplement Figure 3). The phylogenetic tree revealed that *P. tabuliformis* var. *henryi* was closely related to *P. tabuliformis* with strong support. This study provides information on the cp genome of *P. tabuliformis* var. *henryi*, which provides a valuable resource for species identification of Pinaceae and can be used for the identification of closely related species.

## Discussion and conclusions

In terms of morphological structure, *P. tabuliformis* var. *henryi* is typically characterized by smaller fruits with no visible spines on the navel compared to *P. tabuliformis*. The cp genome is a suitable model for phylogenetic studies due to its specificity (Hu et al. 2022). In this study, we assembled the cp genome sequence of *P. tabuliformis* var. *henryi* for the first time and annotated its structure. The results showed no significant differences in genome size, gene content, or order compared to published *P. tabuliformis*, but there were significant differences in GC content (Yu et al. 2017). The phylogenetic results indicated a close relationship between *P. tabuliformis* var. *henryi* and *P. tabuliformis*, suggesting that their provenances were very close. We confirmed the phylogenetic relationships of *P. tabuliformis* var. *henryi* through the cp genome, which could be used to identify and analyze its genetic diversity.

## Supplementary Material

Supplemental Material

Supplemental Material

Supplemental Material

## Data Availability

The genome sequence data that support the findings of this study are openly available in GenBank of NCBI at https://www.ncbi.nlm.nih.gov/ under accession no. MW537659. The associated BioProject, SRA, and Bio-Sample numbers are PRJNA818492, SRR18432540, and SAMN26865303, respectively.
