# Supplementary figures and images for: Complete chloroplast genome sequence of Pinus tabuliformis var. henryi (Mast.) C.T.Kuan 1983 (Pinaceae)
Source: Mitochondrial DNA B Resour. 2024 Jan 11;9(1):83–7. doi: 10.1080/23802359.2023.2301013 (PMC10786436; doi:10.1080/23802359.2023.2301013)

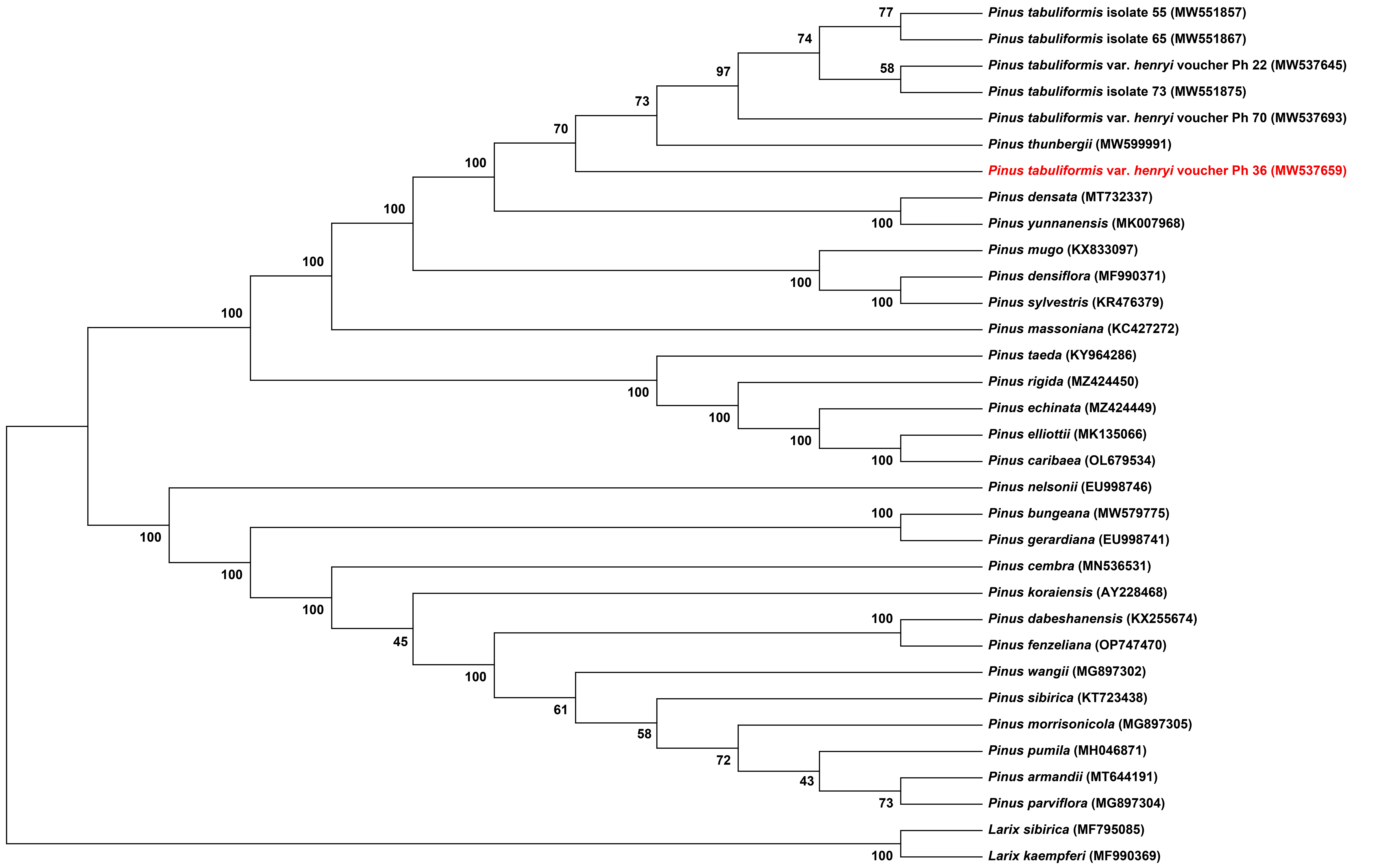

Supplement: Supplemental Material [file TMDN_A_2301013_SM8976.tif]

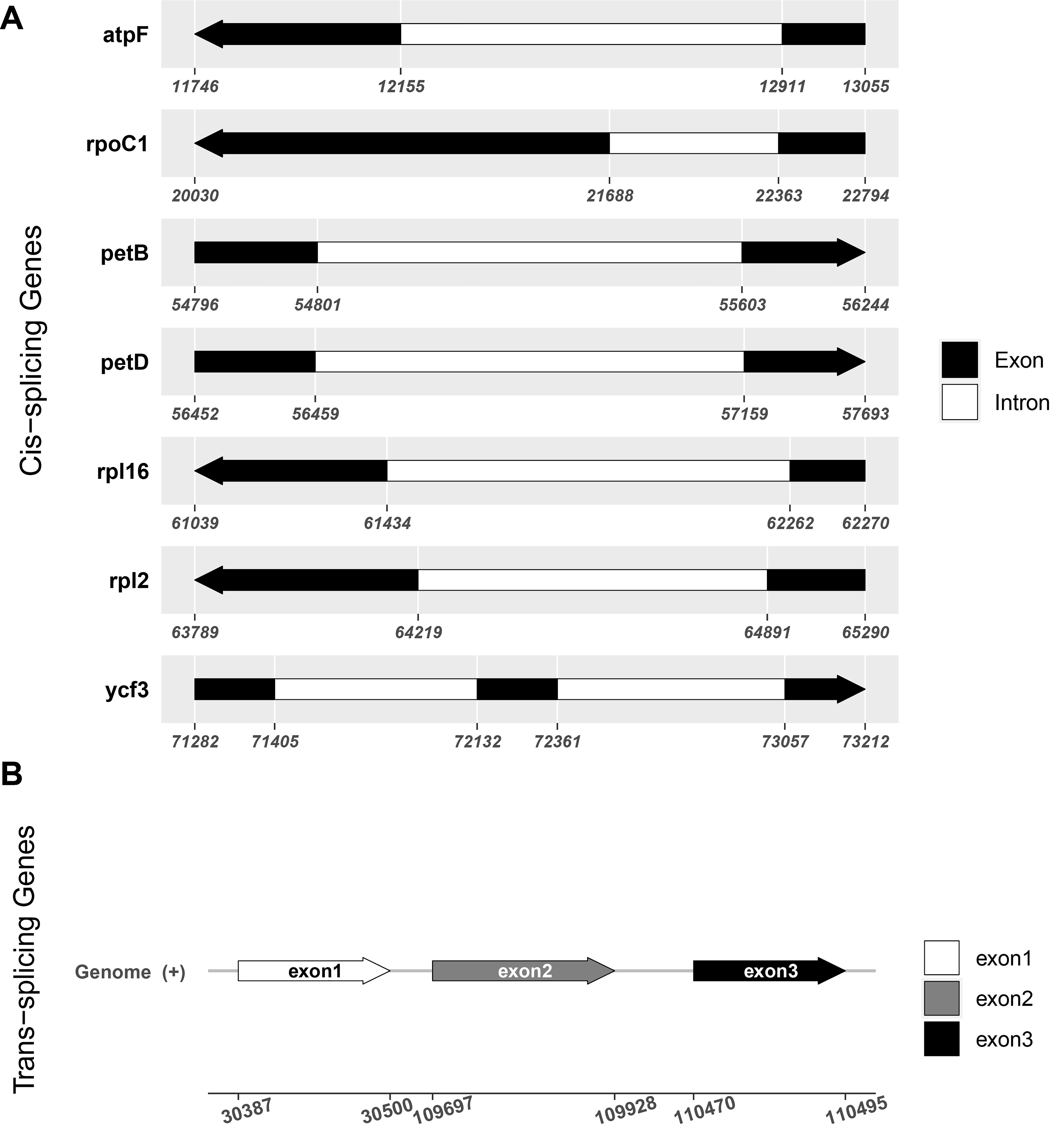

Supplement: Supplemental Material [file TMDN_A_2301013_SM8975.tif]

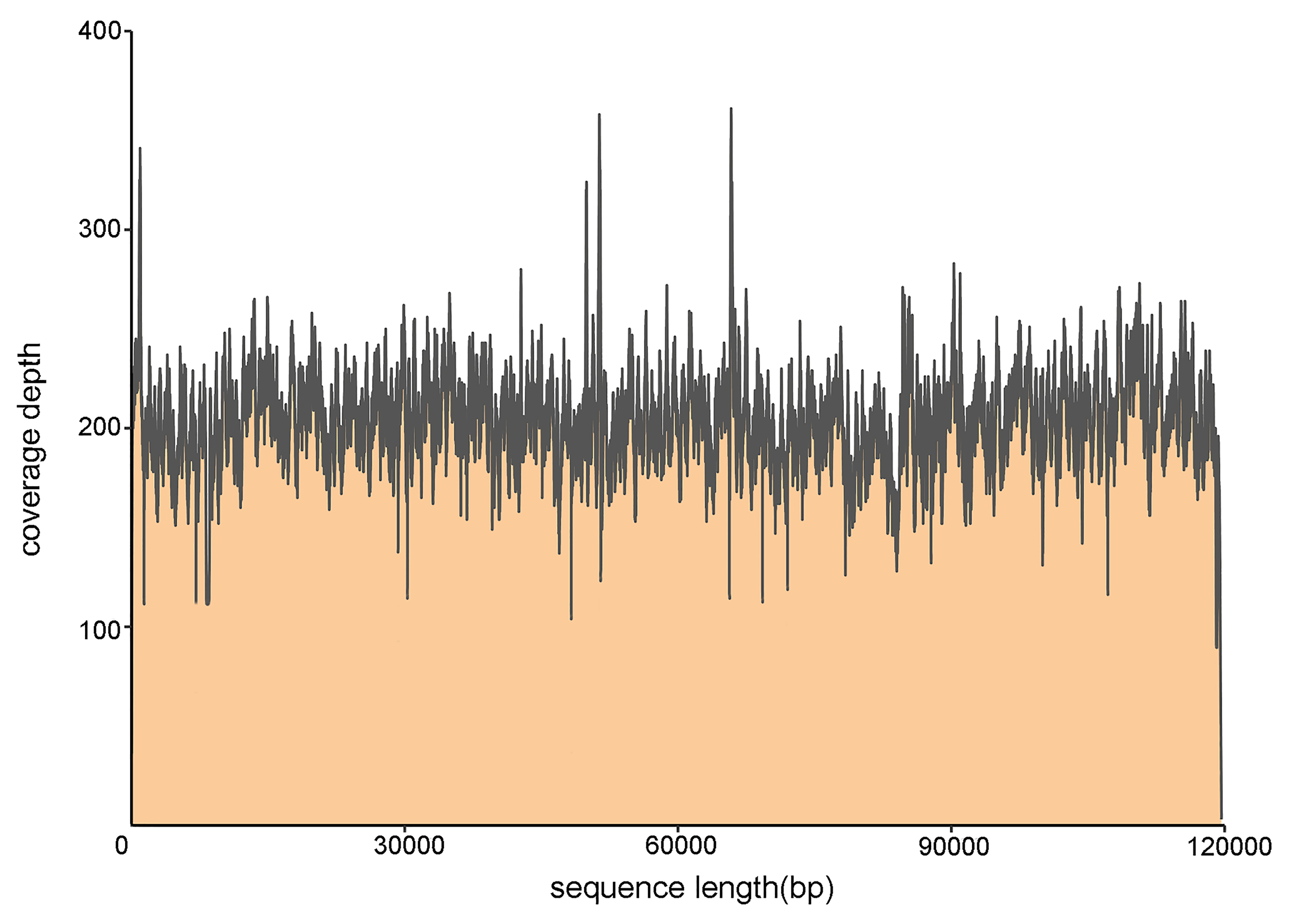

Supplement: Supplemental Material [file TMDN_A_2301013_SM8970.tif]
